# Establishment of Coral–Algal Symbiosis Requires Attraction and Selection

**DOI:** 10.1371/journal.pone.0097003

**Published:** 2014-05-13

**Authors:** Hiroshi Yamashita, Go Suzuki, Sayaka Kai, Takeshi Hayashibara, Kazuhiko Koike

**Affiliations:** 1 Research Center for Subtropical Fisheries, Seikai National Fisheries Research Institute, Fisheries Research Agency, Ishigaki, Okinawa, Japan; 2 Graduate School of Biosphere Science, Hiroshima University, Higashi-Hiroshima, Hiroshima, Japan; Pennsylvania State University, United States of America

## Abstract

Coral reef ecosystems are based on coral–zooxanthellae symbiosis. During the initiation of symbiosis, majority of corals acquire their own zooxanthellae (specifically from the dinoflagellate genus *Symbiodinium*) from surrounding environments. The mechanisms underlying the initial establishment of symbiosis have attracted much interest, and numerous field and laboratory experiments have been conducted to elucidate this establishment. However, it is still unclear whether the host corals selectively or randomly acquire their symbionts from surrounding environments. To address this issue, we initially compared genetic compositions of *Symbiodinium* within naturally settled about 2-week-old *Acropora* coral juveniles (recruits) and those in the adjacent seawater as the potential symbiont source. We then performed infection tests using several types of *Symbiodinium* culture strains and apo-symbiotic (does not have *Symbiodinium* cells yet) *Acropora* coral larvae. Our field observations indicated apparent preference toward specific *Symbiodinium* genotypes (A1 and D1-4) within the recruits, despite a rich abundance of other *Symbiodinium* in the environmental population pool. Laboratory experiments were in accordance with this field observation: *Symbiodinium* strains of type A1 and D1-4 showed higher infection rates for *Acropora* larvae than other genotype strains, even when supplied at lower cell densities. Subsequent attraction tests revealed that three *Symbiodinium* strains were attracted toward *Acropora* larvae, and within them, only A1 and D1-4 strains were acquired by the larvae. Another three strains did not intrinsically approach to the larvae. These findings suggest the initial establishment of corals–*Symbiodinium* symbiosis is not random, and the infection mechanism appeared to comprise two steps: initial attraction step and subsequent selective uptake by the coral.

## Introduction

Reef-building corals engage zooxanthellae, as symbionts that supply them with photosynthetic products, which enable corals to effloresce in oligotrophic tropical seas. This coral–algal symbiosis is a fundamental pillar for biologically and economically important coral reef ecosystems. The symbiont algae, dinoflagellate genus *Symbiodinium* are divided into nine phylogenetically distinct genetic groups (clades A–I) [1], and each clade consists of numerous genotypes (e.g. [2]). Physiological responses to environmental stresses may differ among different clades and genotypes [3], [4]. Sexual progeny of corals can acquire *Symbiodinium* by either of the two modes: vertical transmission (maternal inheritance) or horizontal transmission (acquisition from environment), and corals that acquire *Symbiodinium* from the environment are predominant [5]. The horizontal transmission is considered to be advantageous by enabling corals to acquire *Symbiodinium* adapted to their newly settled environments, however, there is still scant evidence supporting this idea [6]. Furthermore, it is still unclear how corals that acquire *Symbiodinium* by horizontal transmission recognize and acquire their symbionts from the environmental population.

To answer this question, several infection experiments have been carried out using *Symbiodinium* culture strains or freshly isolated cells from adult corals or other zooxanthellate animals [7]–[12]. Although such infections were usually successful, the results often differed between experiments. For example, Cumbo et al. [11] reported that *Acropora* larvae can acquire a wide variety of *Symbiodinium* clades, whereas Yuyama et al. [12] demonstrated that the infectivity of *Symbiodinium* cells in *Acropora tenuis* juveniles can differ among the *Symbiodinium* clades. Thus, it is still unclear whether the new generations of host corals acquire their own *Symbiodinium* randomly or selectively. Previous field observations revealed that in the common reef-building coral *Acropora*, *Symbiodinium* genotype compositions often differ between recruitment/juvenile stages and adult populations [13], [14]. Yamashita et al. [14] only detected clade A and/or D *Symbiodinium* in 55 naturally settled 2-week-old *Acropora* recruits, whereas clade C *Symbiodinium*, which are the dominant symbionts in adult *Acropora* corals, were never detected. The *Acropora* recruits tested by Yamashita et al. [14] were identified to the species level by Suzuki et al. [15] and they comprised at least 10 *Acropora* species, including the dominant *Acropora* species in this area, e.g., *Acropora hyacinthus*, *Acropora digitifera*, *Acropora nasuta*, *Acropora intermedia*, and *Acropora selago*. Thus, it is plausible to suggest that clades A and D *Symbiodinium* play important roles during the initial symbiosis.

In the present study, to clarify whether *Acropora* corals acquire their symbionts selectively or randomly, we compared the *Symbiodinium* genotype compositions (at a finer scale compared with Yamashita et al. [14]) in naturally settled *Acropora* recruits (approximately 2 weeks old) and in the adjacent seawater. Furthermore, we also conducted laboratory experiments in which the *Acropora* coral larvae were artificially infected with naturally occurring densities of various *Symbiodinium* genotypes. Our results demonstrated that in the initial stage of symbiosis, *Acropora* corals had an apparent preference for specific genotypes, even when these genotypes were present at much lower densities than the non-selected *Symbiodinium*. The mechanism of this preferential association was examined by an attraction experiment to determine whether the corals selected the symbionts or whether the *Symbiodinium* selected the corals. Our results from field observations and laboratory experiments suggest underlining two-step mechanisms of attraction and selection for the initial establishment of corals–*Symbiodinium* symbiosis.

## Results

### *Symbiodinium* composition in the environment and natural *Acropora* recruits

**Figure 1:**
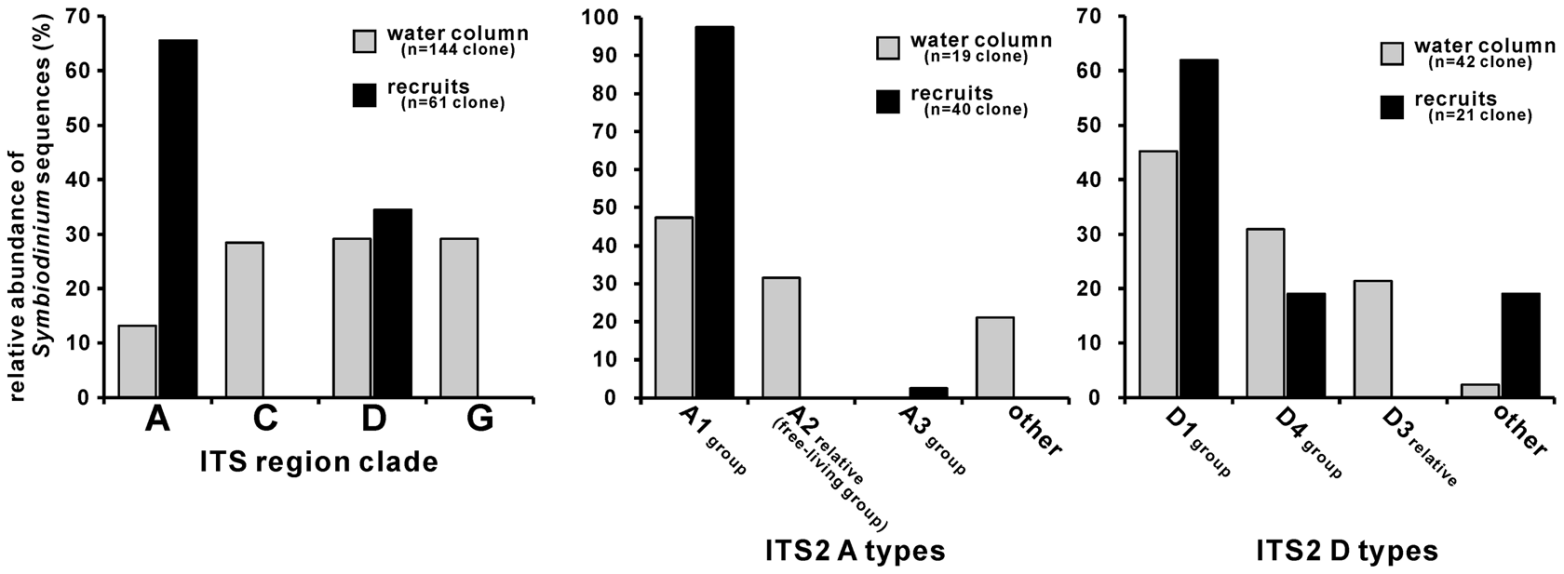
*Symbiodinium* compositions of water column and naturally settled *Acropora* coral recruits. The water samples were collected during the coral mass spawning period in 2011 (gray bars), and the recruits were collected approximately 2 weeks after spawning (black bars). Left histograms show that the clade composition of the recruits did not reflect that of the water column (p<0.05). Clades A and D were detected in both the water and recruit samples, and these *Symbiodinium* clones were further sorted into ITS2 types. Type A1 was the dominant clade A type within the recruits (middle histogram). Right histogram showing clade D ITS2 type compositions; type D1 and D4 group sequences were predominant in both water and recruit samples. Type D3 relative sequences were only detected from water samples. In clade A and D, *Symbiodinium* type compositions were differ between recruits and environments (p<0.05).

*Symbiodinium* genotype composition occurring in the seawater and within naturally settled recruits of *Acropora* corals (approximately 2 weeks after spawning) are shown in Figure 1. A total of 144 environmental *Symbiodinium* DNA clones were obtained from six water samples collected on different dates during a coral spawning period in 2011 (details of the number of DNA clones recovered from each water sample are shown in Table S1). These environmental clones comprised *Symbiodinium* clades A, C, D, and G at 13.19, 28.47, 29.17, and 29.17%, respectively. A total of 61 *Symbiodinium* DNA clones were retrieved from the seven *Acropora* coral recruits. The seven recruits examined were identified as *Acropora intermedia*, *Acropora cytherea*, *Acropora divaricata*, *Acropora hyacinthus*, and *Acropora nasuta/selago* group (three individuals) using molecular markers [16]. These species were common recruits in the study area of Urasoko Bay [15]. Only clade A (65.57%) and D (34.43%) *Symbiodinium* were recovered from these *Acropora* species (details of the number of DNA clones recovered from each recruit sample are shown in Table S1). Therefore, the *Symbiodinium* clade compositions in the recruits did not reflect those in the environment (Fisher's exact tests, p<2.2×10^−16^). At a finer scale grouping based on the ITS2 sequences (ITS2 type groups), clade A environmental clones consisted of type A1 group (47.37%), type A2 relative group (free-living group, 31.58%), and other A types (21.05%). In contrast, clade A within the *Acropora* recruits was limited to two types: type A1 group (97.50%) and type A3 group (2.50%). Therefore, within clade A *Symbiodinium*, type composition within the recruits did not reflect the type composition of the environment (Fisher's exact tests, p = 1.47×10^−6^). Environmental clade D types were type D1 group (45.24%), type D4 group (30.95%), type D3 relative group (21.43%), and other D types (2.38%). Clade D types harbored within *Acropora* recruits consisted of type D1 group (61.90%), type D4 group (19.05%), and other D types (19.05%). Type D3 relative group of *Symbiodinium*, which occurred in the environment, was not detected from natural *Acropora* recruits. Clade D type compositions were statistically different between the environment and recruits (Fisher's exact tests, p = 0.01009).

### Laboratory infection experiments

#### Infection rates and *Symbiodinium* cell densities in the planula larvae

**Figure 2:**
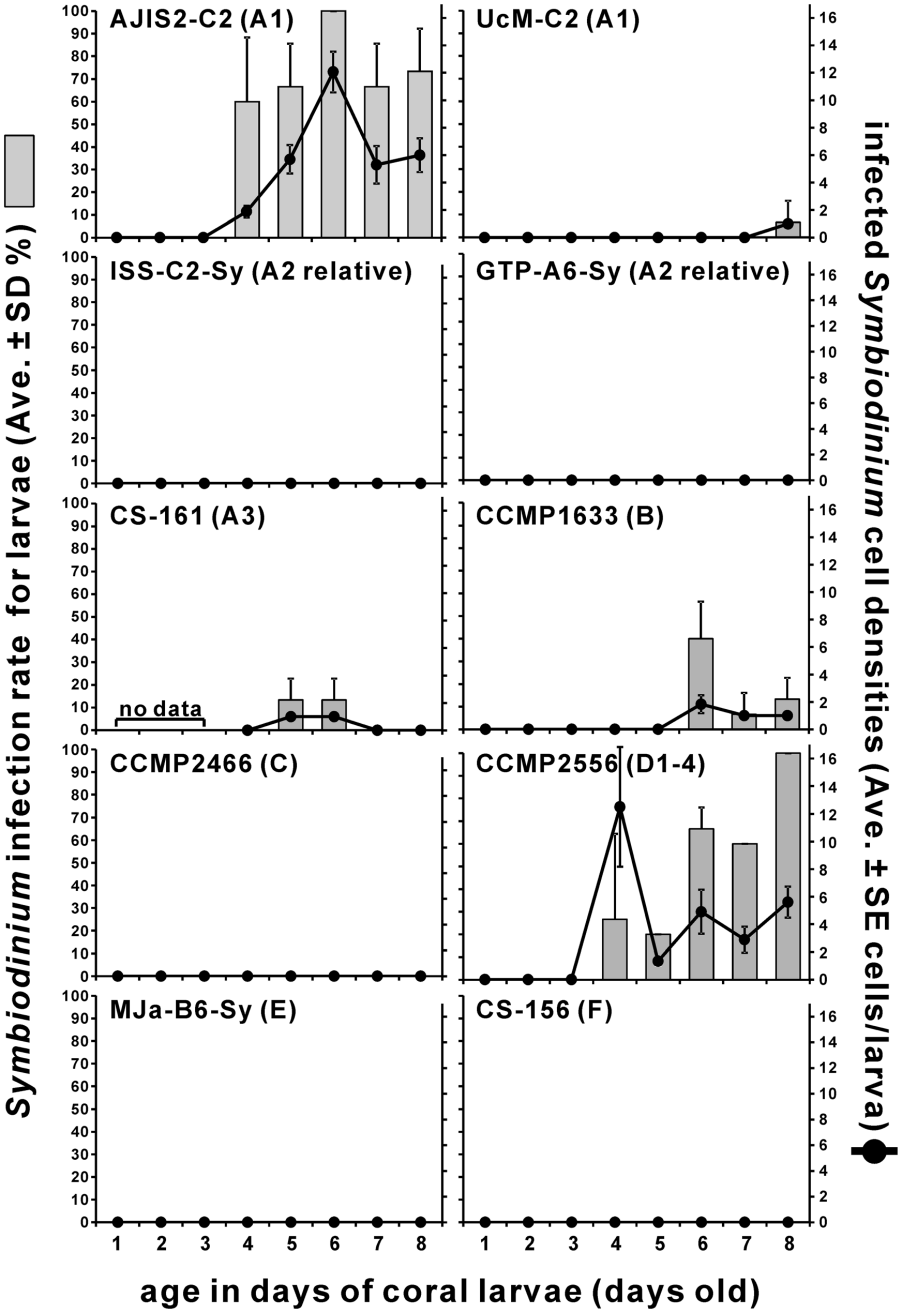
Infection rates and cell densities in laboratory infection tests with apo-symbiotic larvae and 10 *Symbiodinium* culture strains. Bars indicate infection rates (average ± SD of triplicates; left axis), and the lines indicate infected cell densities (average ± SE cells/larva; right axis) for each larval age. *Acropora tenuis* larvae were readily infected with AJIS2-C2 (type A1) and CCMP2556 (type D1-4). Infection rates and infected cell densities of AJIS2-C2 and CCMP2556 cells were significantly higher than that of other *Symbiodinium* culture strains (p<0.05).

We used 10 *Symbiodinium* cultures belonging to clades A–F for the infection test. Five of these successfully infected the *Acropora tenuis* larvae: they were clade A types A1 (strain names  =  AJIS2-C2 and UcM-C2), A3 (CS-161), clade B (CCMP1633), and clade D type D1-4 (CCMP2556) cultures (Figure 2). Among these five cultures, one type A1 culture (AJIS2-C2) and the D1-4 culture (CCMP2556) showed notably higher infection rates (pairwise t-test, p<0.00049 for these two cultures vs. other cultures). All the observed larvae inoculated with AJIS2-C2 and CCMP2556 harbored *Symbiodinium* cells (100% infection rate) on day 6 and 8, respectively. In particular, AJIS2-C2 showed a high infection rate (over 60%) after day 4. The infected cell densities within individual larva were also high with AJIS2-C2 and CCMP2556 cells (pairwise t-test, p<0.00050 for these two cultures vs. other cultures). Maximum cell densities in individual larva (average ± SE cells/larva) were 12.1±1.5 at day 6 for AJIS2-C2 and 12.5±4.3 at day 4 for CCMP2556 (Figure 2). In both cases, total *Symbiodinium* cell numbers in the observed larvae were higher than the inoculated *Symbiodinium* cell numbers. For AJIS2-C2 on day 6, *Symbiodinium* cell division was observed within the larvae, where the observed cell numbers were higher than inoculated cell numbers. For CCMP2556 on day 4, however, four larvae acquired *Symbiodinium* cells, and the numbers of cells within the larvae were 5, 10, 10, and 25; thus, all the inoculated cells might be acquired by the larvae (average = 12.5 cells). The maximum infected cell densities in the larvae were low with type A3 (CS-161), other type A1 culture (UcM-C2), and clade B culture (CCMP1633), with 1±0 at days 5 and 6, 1±0 at day 8, and 1.8±0.7 cells/larva at day 6, respectively. Although AJIS2-C2 and UcM-C2 belong to the same type A1 *Symbiodinium* group, their infection rates and infected cell densities were significantly different.

#### Infection rates and cell densities with different inoculated cell densities

The cells of one type A1 culture (AJIS2-C2) and type D1-4 (CCMP2556) culture were acquired by the larvae even at a low cell density (7 cells/cup = 1 cell/larva = 140 cells/L), whereas ISS-C2-Sy (type A2 relative) was acquired by the larvae only at the highest cell density (700 cells/cup = 100 cells/larva = 14,000 cells/L; Table 1). Infection rates (average ± SD) and infected-cell densities (average ± SE cells/larva) with one type A1 (AJIS2-C2) were 26.7±11.5% and 1.8±0.5 cells/larva at a low cell density, 46.7±34.0% and 1.1±0.1 cells/larva at a medium cell density (70 cells/cup = 10 cells/larva = 1400 cells/L), and 100±0% and 4.3±0.8 cells/larva at a high cell density. Infection rates and infected-cell densities with type D1-4 (CCMP2556) were 20.0±16.3% and 1.0±0 cells/larva at a low cell density, 73.3±24.9% and 2.0±0.3 cells/larva at a medium cell density, and 86.7±18.9% and 10.9±3.5 cells/larva at a high cell density.

**Table 1:** Infection rates and infected *Symbiodinium* cell densities with different inoculated cell densities are shown.

Type	Strain	Inoculated cell density	Infection rate (%)(a)	Infected-cell density (cells/larva)(b)
A1	AJIS2-C2	Low	26.7±11.5	1.8±0.5
		Medium	46.7±34.0	1.1±0.1
		High	100±0	4.3±0.8
A2 relative	ISS-C2-Sy	Low	0	0
		Medium	0	0
		High	6.7±9.4	1±0
D1-4	CCMP2556	Low	20.0±16.3	1.0±0
		Medium	73.3±24.9	2.0±0.3
		High	86.7±18.9	10.9±3.5

Inoculated *Symbiodinium* cell densities were low (7 cells/cup = 1 cell/larva = 140 cells/L), medium (70 cells/cup = 10 cells/larva = 1400 cells/L), and high (700 cells/cup = 100 cells/larva = 14,000 cells/L).

(a) Infection rates are the average ± SD of triplicate experiments.

(b) Infected-cell densities are the average ± SE in infected larvae.

### Attraction behaviors of the larvae and *Symbiodinium*

The larvae swam around in a random manner, but they never stopped at specific spots on filters at where 10 *Symbiodinium* cultures were applied; thus, we concluded that the larvae were not attracted to any of the *Symbiodinium* cell spots. However, *Symbiodinium* cells of one type A1 culture (AJIS2-C2), type A2 relative (free-living group; ISS-C2-Sy), clade B (CCMP1633), type D1-4 (CCMP2556), and clade E (MJa-B6-Sy) were found within the larval-containing PCR tubes (Table 2). Five AJIS2-C2 cells and 12 CCMP2556 cells were observed in five of 12 larvae and seven of 15 larvae, respectively. Although a total of 15 larvae (3 larvae/tube ×5 tubes) were prepared for each strain, a few larvae died after 24 h; therefore, some observed larvae numbers were less than the initial number. Furthermore, two ISS-C2-Sy (type A2 relative) cells, CCMP1633 (clade B), and CCMP2556 (type D1–4), and five cells of MJa-B6-Sy (clade E) were found within the water in tubes with the larvae. In the control tubes (without the larvae), only one cell of CCMP2466 (clade C) was found, although we observed 40 control tubes for the eight culture strains. Thus, these cultured cells which was observed within larval-containing PCR tubes, moved through the nylon mesh at the tube openings, indicating that they were attracted to the larvae in tubes and probably not freely diffusing. The cell numbers of AJIS2-C2, CCMP2556, and MJa-B6-Sy were significantly higher in the tubes with larvae (Δdeviance = 6.93, Δdf = 1, p = 0.00847; Δdeviance = 19.4, Δdf = 1, p = 1.06×10^−5^; Δdeviance = 6.93, Δdf = 1, p = 0.00847, respectively). In contrast, the cell numbers of ISS-C2-Sy and CCMP1633 were somewhat higher in the tubes with larvae, but the difference was not significant (Δdeviance = 2.77, Δdf = 1, p = 0.0959; Δdeviance = 2.77, Δdf = 1, p = 0.0959, respectively). Moreover, in the case of CS-161, CCMP2466, and CS-156, the cell numbers in the tubes with and without larvae were not significantly different (Δdeviance = 0, Δdf = 1, p = 1; Δdeviance = 1.39, Δdf = 1, p = 0.239; Δdeviance = 0, Δdf = 1, p = 1, respectively).

**Table 2:** *Symbiodinium* cell numbers observed in the attraction tests using PCR tubes with and without *Acropora tenuis* larvae.

Clade/type	Culture name	Tube no.	*Symbiodinium* cell number		
			Tubes with larvae		Tubes without larvae
			larvae(a)	water	water (control)
A1	AJIS2-C2	1	1 ^(1/2)^	0	0
		2	3 ^(3/3)^	0	0
		3	0 ^(0/3)^	0	0
		4	1 ^(1/3)^	0	0
		5	0 ^(0/1)^	0	0
A2 relative	ISS-C2-Sy	1	0 ^(0/3)^	0	0
		2	0 ^(0/3)^	0	0
		3	0 ^(0/3)^	1	0
		4	0 ^(0/3)^	0	0
		5	0 ^(0/3)^	1	0
A3	CS-161	1	0 ^(0/3)^	0	0
		2	0 ^(0/2)^	0	0
		3	0 ^(0/3)^	0	0
		4	0 ^(0/2)^	0	0
		5	0 ^(0/3)^	0	0
B	CCMP1633	1	0 ^(0/3)^	0	0
		2	0 ^(0/3)^	0	0
		3	0 ^(0/3)^	2	0
		4	0 ^(0/3)^	0	0
		5	0 ^(0/1)^	0	0
C	CCMP2466	1	0 ^(0/3)^	0	0
		2	0 ^(0/3)^	0	0
		3	0 ^(0/3)^	0	0
		4	0 ^(0/1)^	0	1
		5	0 ^(0/3)^	0	0
D1-4	CCMP2556	1	1 ^(1/3)^	0	0
		2	1 ^(1/3)^	0	0
		3	4 ^(2/3)^	1	0
		4	5 ^(2/3)^	0	0
		5	1 ^(1/3)^	1	0
E	MJa-B6-Sy	1	0 ^(0/3)^	1	0
		2	0 ^(0/3)^	0	0
		3	0 ^(0/3)^	1	0
		4	0 ^(0/2)^	3	0
		5	0 ^(0/2)^	0	0
F	CS-156	1	0 ^(0/2)^	0	0
		2	0 ^(0/2)^	0	0
		3	0 ^(0/2)^	0	0
		4	0 ^(0/3)^	0	0
		5	0 ^(0/2)^	0	0

(a) The infected larvae/total observed larvae are shown in parentheses.

## Discussion

Our results clearly showed that *Acropora* corals do not randomly acquire *Symbiodinium* from surrounding environments, at the initial stage of their symbiosis. Environmental *Symbiodinium* (i.e., solitary *Symbiodinium* in the environment away from host animals) are highly diverse. Clades A, B, C, D, E, G, and H have been previously detected from the water column and/or sediment samples in the Pacific [14], [17]–[19]. In the present study, we collected water samples during the coral spawning period of 2011 to clarify potential symbiont sources for the coral larvae, and detected clades A, C, D, and G. Therefore, it was plausible that apo-symbiotic corals acquired these diversified environmental *Symbiodinium*. In our study area, Urasoko Bay, several species of adult *Acropora* corals, *Porites lutea*, *Cyphastrea serailia* harbored only clade C *Symbiodinium* [14], [20], whereas *Pocillopora eydouxi* and *Favites abdita* harbored mainly clade C with background level of clade D *Symbiodinium* [20]. The environmental *Symbiodinium* populations in Urasoko Bay were previously reported to primarily consist of clade C, and clades A/D were relatively rare [14]. In the present study, however, almost equal number of clade C and D clones was recovered from water samples. Because ITS1 copy number of clade D *Symbiodinium* was estimated to be approximately three times higher that of clade C [21], it is plausible to assume that clade C populations were approximately three times larger than clade D populations in the same period. Nevertheless, our present and previous field observations [14] demonstrated that naturally settled 2-week-old *Acropora* coral recruits usually harbored clades A and/or D *Symbiodinium*, which were rare in the environment. Littman et al. [22] reported that high cell densities (1000–4000 cells/mL) of *Symbiodinium* were present in the benthic communities of the Great Barrier Reef; furthermore, in Hawai‘i, Takabayashi et al. [18] demonstrated that the diversity of *Symbiodinium* was higher in sediments than that in the water column. Thus, sediments are considered to be an important source of symbionts for corals. In our study area of Urasoko Bay, *Symbiodinium* clade compositions in the sediments were previously reported [14]. However, *Symbiodinium* cell densities could not be determined due to the low *Symbiodinium* DNA concentrations in the sediment samples; the dominant *Symbiodinium* in the sediments belonged to clade C, whereas clade A and D were relatively rare, as found in the water column [14]. This result may indicate that the benthic *Symbiodinium* cell densities and diversity in our study area of Urasoko bay were lower than those in other coral reef environments.

In previous field observations, clades A and/or D *Symbiodinium* were also detected from *Acropora* recruits, e.g., at the Great Barrier Reef [13], [23], [24] and Ishigaki Island [14]. In the present study, we only examined seven *Acropora* recruits from five *Acropora* species; *Symbiodinium* clade compositions within the recruits were in accordance with our previous observation in the same area [14]. Furthermore, ITS2 type analysis revealed that clade A type compositions were completely different between the environment and the recruits. Environmental A types consisted of type A1, A2 relative (free-living), and other A type group, whereas naturally settled recruits primarily harbored type A1 and a few A3 group members. Type A2 relative group was not detected in natural recruits. This result indicated that *Acropora* corals may engage in symbiosis with specific types in the specific clade. In contrast, clade D types within the recruits were not so different from environmental D types (but statistically different). Many clade D types have been determined by PCR–denaturing gradient gel electrophoresis (DGGE) fingerprinting, and several types are known to have two or three different ITS2 sequences within their genomes [25]. For example, type D1-4 (formerly type D1a) has both a D1 sequence and a D4 sequence in its genome [25]. PCR cloning separately detects D1 and D4 sequences. Therefore, D1 and D4 sequences recovered in our study were presumed to originate from a *Symbiodinium* cell with D1 and/or D4 sequence(s) in its genome. D1 and D4 were the most abundant sequences in both the water and recruit samples; thus, the clade D type compositions were not so different between the samples (p = 0.01009), compared to the case of A type compositions (p = 1.47×10^−6^). However, type D3 relative *Symbiodinium* was only detected from water samples and never from recruit samples. This result indicates that *Acropora* corals do not randomly acquire environmental clade D *Symbiodinium*, as is the case with clade A *Symbiodinium*.

We performed infection tests with apo-symbiotic planula larvae of *Acropora tenuis* using clade A–F *Symbiodinium* culture strains. Infection test results well reflected field observations, particularly type A1 (AJIS2-C2) and type D1-4 (CCMP2556) were readily acquired by *A. tenuis* larvae. Interestingly, type A1 strain UcM-C2, with an ITS region sequence identical to that of AJIS2-C2 [19], was not often acquired by the larvae. This result may indicate that characteristics may differ even within the same ITS2 sequence. In the present study, although *A. tenuis* larvae were used in the laboratory infection tests, we could not find naturally settled *A. tenuis* recruits in our field samples. Two of 55 2-week-old *Acropora* recruits that were analyzed previously by Yamashita et al. [14] were identified as *A. tenuis* by Suzuki et al. [15], and these corals harbored clade D *Symbiodinium*. In the present study, however, the laboratory infection test revealed that *A. tenuis* larvae can acquire clade A (type A1) and clade D (type D1-4) strains. Type A3 and clade B *Symbiodinium* strains also infected the larvae, although the infection rates were lower than that for types A1 and D1-4. Previous laboratory experiments reported that type A3 and clade B *Symbiodinium* were acquired by apo-symbiotic *Acropora* larvae/juveniles [12], [26]. Although, we could not detect type A3 *Symbiodinium* in the water samples, one A3 clone was recovered from a natural recruit. Environmental type A3 *Symbiodinium* was detected by Yamashita and Koike [19] in water samples collected from Urasoko Bay. In contrast, clade B *Symbiodinium* was never detected from environmental waters or *Acropora* corals of Urasoko Bay [14], [20]. Furthermore, type A2 relative and clade C *Symbiodinium* were readily detected in the water column of Urasoko Bay; however, these groups were never acquired by *Acropora tenuis* larvae. These results are in agreement with previous reports that type A2 relative groups were not acquired by cnidarian hosts [27], [7] and that clade C *Symbiodinium* was relatively rare in *Acropora* recruits from the field [13], [14]. In our trial, type A2 relative *Symbiodinium* strain ISS-C2-Sy could infect the larvae under a high cell density condition (14,000 cells/L), whereas at low and medium cell densities (140 or 1400 cells/L), the larvae did not acquire ISS-C2-Sy cells. This result may indicate that the larvae could acquire any *Symbiodinium* if the cells were abundant. However, in coral reef environments, *Symbiodinium* cell densities in the water column were usually only several hundred cells/L or less [14], although higher cell densities have been reported from the Great Barrier Reef [22]. According to previous laboratory infection tests, *Acropora* larvae can acquire several *Symbiodinium* genotypes (e.g. [11]), whereas Yuyama et al. [12] demonstrated that some *Symbiodinium* clades did not infect *Acropora* juveniles. Laboratory infection tests have revealed important information regarding the initial symbiotic stage of *Symbiodinium* and corals, e.g., the timing of symbiont acquisition by the coral larvae [9], [10], differential growth rates [23], and gene expression [28] among juvenile polyps infected with different *Symbiodinium*. However, these previous experiments required high-density *Symbiodinium* cells, and thus might be not representative of natural infection behavior.

Although the cell densities of symbiont source were low, corals established symbiosis with these sources in the field. Furthermore, types A1 and D1-4 cells were successfully acquired by the larvae at lower cell densities (140 cells/L). It is plausible to assume that corals and/or *Symbiodinium* attract each other to establish initial symbiosis. In our attraction tests, the *A. tenuis* larvae were not attracted by any of the *Symbiodinium* cultures, whereas five of eight tested *Symbiodinium* strains were found within the tubes with *A. tenuis* larvae. That is, type A1 (AJIS2-C2), type A2 relative (ISS-C2-Sy), clade B (CCMP1633), type D1-4 (CCMP2556), and clade E (MJa-B6-Sy) *Symbiodinium* cultures. Among these five strains, type A1, type D1-4, and clade E *Symbiodinium* cell densities were significantly higher in the tubes with larvae compared with that in the tubes without larvae. Thus, these three *Symbiodinium* strains are considered to have been attracted by the larvae. We used five string tubes in this attraction test, thus the possibility of a disproportionate distribution of *Symbiodinium* cells could not be discounted entirely. However, we found that 5–14 AJIS2-C2, CCMP2556, and MJa-B6-Sy cells were present in the tubes with larvae, which did not support this possibility, because we did not find any *Symbiodinium* cells in the tubes without larvae (control tubes). The attraction behavior of *Symbiodinium* cells has also been reported in previous studies. For example, in the laboratory experiments, motile *Symbiodinium* cells exhibited taxis for the coral mouth [29], and some of the coral extracts can attract *Symbiodinium* cells (Takeuchi et al. personal communication). Indeed, motile-stage *Symbiodinium* have chemosensory responses [30], and also they exhibit phototaxis toward green light [31]. Some of the corals, green fluorescence was concentrated around the mouth region of the larvae approximately 4–6 days after spawning [32], which corresponded to the timing of symbiont acquisition by the coral larvae. In the environment, these abilities and/or characteristics of *Symbiodinium* and corals may be used for initial contact in the establishment of symbiosis. Hollingsworth et al. [31], [32] generated the “beacon hypothesis,” suggesting that green fluorescent proteins may play a role in attracting motile *Symbiodinium* cells to apo-symbiotic larvae. In fact, motile-stage *Symbiodinium* cells have an eyespot that is considered to reflect green light [33]. In our preliminary experiment, dead *Symbiodinium* cells were not acquired by the larvae (Text S1). Although dead cells and live non-motile cells may be different when the larvae acquire *Symbiodinium* cells, it is inferred also from this preliminary data that attraction behavior of *Symbiodinium* cells is one of the key functions for initiating the symbiosis. Our laboratory and field observations, however, indicated that *Acropora* corals do not acquire all types of attracted *Symbiodinium* cells. Type A2 relative, clade B, and E *Symbiodinium* cells were found within the tubes with larvae, but they did not infect the larvae in test tubes, and these genotypes were not found in the field coral recruits/juveniles. Furthermore, even with the readily acquired types A1 and D1-4, cell densities within the larvae were independent of the inoculated cell densities. In the present study, we inoculated type A1 and D1-4 *Symbiodinium* at different densities that ranged from 1 to 100 cells/larva. The *A. tenuis* larvae successfully acquired *Symbiodinium* cells even with a low *Symbiodinium* cell density (1 cell/larva). However, even when we inoculated high densities (100 cells/larva) of type A1 and D1-4 *Symbiodinium*, the larvae only harbored 4.3±0.8 and 10.9±3.5 cells of type A1 and D1-4, respectively. These results indicate that the initial symbiosis between corals and *Symbiodinium* is not determined by only infectivity of the *Symbiodinium*, and possibly involves selection by the corals. Several reports have indicated that corals recognize *Symbiodinium* cell-surface sugars (glycoproteins and glycolipids) by means of lectin at the onset of symbiosis [34]-[37]. Thus the initial establishment of symbiosis between apo-symbiotic corals and *Symbiodinium* cells involves a two-step selection; initial attraction step and subsequent selective uptake step.

Both types A1 and D1-4 *Symbiodinium* groups have been detected from various hosts from various geographic ranges [38], [25]. In addition, the combined results of our field observations and laboratory experiments suggested that type A1 and D1-4 *Symbiodinium* are the first symbiont partners of *Acropora* corals. Thus, a future challenge is to elucidate the benefits of symbiosis establishment between *Acropora* corals and type A1/D1-4 *Symbiodinium*.

## Materials and Methods

No specific permissions were required for collection of sea water samples in the study area. Sampling of corals was exceptionally permitted by the Okinawa Prefectural Government for research use (No. 23-47).

### *Symbiodinium* and newly recruited *Acropora* corals collected from the environment

To determine the environmental *Symbiodinium* composition during the coral spawning period, when newly released coral larvae and/or recruits would be expected to acquire their symbionts, we collected 3-L water samples from a 3-m depth (immediately above after-mentioned artificial settlement plates) once a day from the middle of Urasoko Bay, Ishigaki Island, Okinawa, Japan (24°27′ N, 124°13′ E) on May 16, 17, 18, 19, 20, and 25, 2011. Slick of coral egg/gametes by natural spawning was observed on the morning of May 16, 17, and 18, 2011. In this study area, the naturally settled *Acropora* recruits were dominated by *Acropora digitifera*, *Acropora tenuis*, *Acropora nasuta*, *Acropora hyacinthus*, *Acropora loripes*, *Acropora divaricata*, *Acropora selago*, *Acropora intermedia*, and *Acropora gemmifera* [15]. Water samples were subjected to DNA extraction according to the method of Yamashita et al. [14]. The seven individuals of newly recruited *Acropora* corals (recruits) were randomly collected from artificial settlement plates (same design as Suzuki et al. [15]) placed at the bottom of the water collection site. The plates were retrieved on June 3, 2011, approximately two weeks after natural spawning. DNA extraction and molecular species identification were performed according to the method described by Suzuki et al. [16] using the recruit samples.

### PCR, cloning, and sequencing of *Symbiodinium* in the water and recruit samples

The internal transcribed spacer (ITS) region (the entire ITS1-5.8S rRNA-ITS2 gene region) sequences of *Symbiodinium* from the water column and recruits were amplified by means of *Symbiodinium* specific primer set “r18Sf” and “Sym28Sr” described by Yamashita and Koike [19]. The amplicons were cloned using a pCR4-TOPO TA cloning kit (Invitrogen, Carlsbad, CA, USA). Then sequencing were performed by Macrogen Japan (http://www.macrogen-japan.co.jp/), namely, the sequencing reactions were performed in a BioRad DNA Engine Dyad PTC-220 Peltier Thermal Cycler using a ABI BigDye Terminator v3.1 Cycle Sequencing Kits with AmpliTaq DNA polymerase (FS enzyme) (Applied Biosystems), and the fluorescent-labeled fragments were subjected to electrophoresis in an ABI 3730xl sequencer (Applied Biosystems). In total, 25 clones from each water sample and 10 clones from each recruit sample were sequenced. These sequences obtained in the present study were deposited in GenBank/EMBL/DDBJ (accession numbers AB849693-AB849897).

### Grouping of *Symbiodinium* DNA clones from seawater and recruits

Initially, all DNA clones (144 from seawater and 61 from recruits) were divided into clades by BLAST search via the website of the DNA Data Bank of Japan (DDBJ; http://www.ddbj.nig.ac.jp/index-j.html). Clones of clades A and D, obtained from both seawater and recruits, were further classified into ITS2 type. The clade A and D sequences determined in the present study were aligned with ITS2 type reference sequence files downloaded from the website GeoSymbio [39] (https://sites.google.com/site/geosymbio/home) using MEGA Version 5 [40]. The alignment files, including our clade A and D sequences, are available on request from the corresponding author. A cloning method was used for *Symbiodinium* identification, so we could not determine whether a few nucleotides changes were attributable to PCR errors or sequence variants. Thus, we conservatively categorized our sequence data into “type group” that comprised sequences sharing 100% identity with known (published) ITS type sequences, and closely related sequences. In the present study, we categorized type A1, type A2 relative, and type A3 groups, where these groups were based on the sequences of accession number AF427466 (from culture Cx), AF427468 (a free-living *Symbiodinium* group from culture Zs), and AF427467 (from culture T), respectively. In clade D, type D1 group in the present study was based on the sequence EU449061, type D4 group was based on AF499802, and type D3 relative group was based on AY686650. It should be noted that some of the *Symbiodinium* cells have two or three different ITS2 sequences within their genomes, for instance type D1-4 (formerly type D1a) *Symbiodinium* have D1 sequence and D4 sequence [25]. The cloning technique used in the present study individually recovers D1 sequence and D4 sequence from type D1-4 *Symbiodinium*.

### Laboratory infections

#### *Symbiodinium* culture strains

The *Symbiodinium* cultures used in this study (total 10 cultures) are listed in Table S2. The cultures CCMP1633 (clade B), CCMP2466 (clade C), and CCMP2556 (clade D, type D1-4) were purchased from the Provasoli–Guillard National Center for Culture of Marine Algae and Microbiota (ME, USA) and CS-156 (clade F) and CS-161 (clade A, type A3) were purchased from the Commonwealth Scientific & Industrial Research Organization (VIC. Australia). The other cultures; AJIS2-C2, UcM-C2 (both are clade A, type A1), GTP-A6-Sy, ISS-C2-Sy (both are clade A, type A2 relative) and MJa-B6-Sy (clade E), were originally isolated by Yamashita and Koike [19]. All cultures were maintained in a 27°C incubator under a light regime of 80–120 µmol photon m^−2^ sec^−1^ (12-h light/dark period) in IMK medium (Sanko Jyunyaku, Tokyo, Japan). Among these culture strains, type A2 relative, type A3 and clades B, E, and F exhibited rapid growth by cell division compared with *Symbiodinium* type A1 and clades C and D, during the logarithmic growth phase (Yamashita, personal observation). Thus, the *Symbiodinium* cell densities could have differed among the culture strains if logarithmic growth phase cells had been used for the infection tests because of their different growth rates. To avoid cell divisions during the experiment periods (24 h) for infection, we used stationary growth phase cultures. During this phase, usually the cells can transform into motile form (can swim) at daytime, however, cell divisions occur rarely.

#### Preparation of apo-symbiotic planula larvae

Seven parental *Acropora tenuis* colonies were collected from Urasoko Bay on June 23, 2011. The colonies were maintained in running seawater for several days. Spawning was artificially induced with H_2_O_2_ [41], and the egg-sperm bundles released by these corals were collected in a single 5-L container. Artificially fertilized eggs were washed to remove any remaining sperm and transferred to 0.4-µm-filtered offshore seawater (FSW) in several 1-L containers (egg/planula densities of approximately 500 L^−1^). The stock larvae were maintained in a 27°C incubator, as described above, where the FSW was changed every 1–2 days. Planula larvae were randomly selected from these larval stock tanks and used in the infection tests.

#### Infection tests using *Symbiodinium* culture strains and apo-symbiotic planula larvae

All infection tests were performed in 100-mL glass cups (columnar form; inner diameter = 47 mm, height = 54 mm). Seven randomly selected apo-symbiotic larvae from larval stock tanks were combined with 50 cells of each *Symbiodinium* strain in 50 mL of FSW. Each combination was performed in triplicates. After 24 h in the 27°C incubator, five larvae were randomly selected from each cup and observed the presence of *Symbiodinium* using an epifluorescent microscope (BX50, Olympus, Tokyo, Japan; UV ex.). Individual larvae were gently squashed between a glass slide and cover slip, and the autofluorescence of *Symbiodinium* chlorophyll *a* within larval body was observed. In the infected larvae, *Symbiodinium* cell numbers within each larva were manually counted by eye. The infection rate (%) in each cup was calculated as the number of infected larvae/number of observed larvae ×100. The experiment started at 20 h after fertilization. At this time, the corals were still at the embryo stage (not at the larval stage), but Montgomery and Kremer [42] reported that the scyphozoan *Linuche unguiculata* can acquire their symbionts by the 128-cell stage. Thus, we started the infection test at 20 h after fertilization. The experiment was repeated daily until the larvae were 8 days old, i.e., the observations, larvae and *Symbiodinium* combinations were set up each day. A schematic diagram of the infection test procedure is shown in Figure S1.

#### Infection tests with different inoculated *Symbiodinium* cell densities

To determine whether infection rates and infected cell densities within the larvae were dependent on inoculated *Symbiodinium* density, we repeated the above infection experiments with 7 larvae and 7, 70, and 700 cells per cup of type A1 (AJIS2-C2) and type D1-4 (CCMP2556) strains, which showed the highest infectivity, and type A2 relative (ISS-C2-Sy), which was never acquired by the larvae in the above experiments. These cultures were supplemented in densities of 7, 70, and 700 cells per cups, and each cup contained seven larvae in total. Therefore, each larva had the potential to be infected with 1, 10, or 100 *Symbiodinium* cells. All experimental combinations were performed in triplicates. The larvae were examined by epifluorescent microscopy 24 h after infection. This experiment was performed in 2012 (*A. tenuis* larvae were maintained in the same manner as that in the 2011 experiment period).

### Attraction tests

Attraction tests were performed to examine migration of the larvae to *Symbiodinium* and of *Symbiodinium* to the larvae. The 10 *Symbiodinium* strains were filtered through Whatman GF/C filter paper (φ = 47 mm; GE Healthcare, UK) to randomly trap 1000 cells of each strain as a small spot on the paper. Spots without *Symbiodinium* cells were prepared as controls. Three spots were prepared for each *Symbiodinium* culture and for the control. In total 30 spots of *Symbiodinium* cells and three control spots without cells were randomly arranged on 11 GF/C filters. These filters were arranged in a single layer in a square container filled with 1 L of FSW, and 100 *Acropora tenuis* larvae were released into the container. After 1, 3, 6, 12, 24, and 48 h, we visually examined whether the larvae were attracted to *Symbiodinium* cell spots.

To examine the attraction of *Symbiodinium* to the larvae, three *A. tenuis* larvae each were placed into small plastic tubes (five string tubes cutting from 8-strips PCR tubes; 280-µL capacity), and tops of the tubes were covered with 200-µm nylon mesh (larvae cannot pass through, but *Symbiodinium* cells can pass through). Five-string tubes and additional five-string tubes without the larvae were placed together into plastic containers with 500 mL of FSW. Cells (500 each) of the *Symbiodinium* strains type A1 (AJIS2-C2), type A2 relative (ISS-C2-Sy), type A3 (CS-161), clade B (CCMP1633), clade C (CCMP2466), type D1-4 (CCMP2556), clade E (MJa-B6-Sy), and clade F (CS-156) were added to the containers. After 24 h, the tubes were carefully removed from each container, and the contents (larvae and seawater) were observed by epifluorescent microscopy for presence/absence of *Symbiodinium* cells.

### Statistical analyses

All of the statistical tests were performed by using R version 2.15.2 [43]. Frequencies of *Symbiodinium* clades and types recovered from seawater and recruits were analyzed using Fisher's exact tests. Comparisons were made between the *Symbiodinium* composition of recruits and seawater. In this analysis, the *Symbiodinium* composition of seawater was defined as the expected value. Null hypothesis (*H_0_*) was that the *Symbiodinium* composition within the recruits would resemble that of the seawater (expected value). For the infection tests, Kruskal–Wallis tests were performed for differences among the inoculated *Symbiodinium* cultures. Maximum infection rates and cell numbers at the day of maximum infection were used for these analyses. Significant differences were analyzed by pair-wise comparison using Holm correction t-test. For attraction test, we performed a log-likelihood ratio test based on the generalized linear model (GLM) for each *Symbiodinium* strain to examine whether the cell numbers of the strain significantly changed between the tubes with and without larvae. The statistical test assumed the Poisson distribution of *Symbiodinium* cells and adopted a log-link function. The significance probability in this test was calculated with the GLM function in R. In the present study, less than 0.05 p-values were considered as statistically significant.

## Supporting Information

Figure S1 A schematic diagram of the infection test procedure.
(TIF)

Table S1 Number of *Symbiodinium* DNA clones recovered from water and *Acropora* recruit samples.
(DOCX)

Table S2 *Symbiodinium* culture strains used for infection tests are listed.
(DOCX)

Text S1 Infection test using apo-symbiotic *Acropora tenuis* larvae and dead *Symbiodinium* cells.
(DOCX)
