# Supplementary figures and images for: Establishment of Coral–Algal Symbiosis Requires Attraction and Selection
Source: PLoS One. 2014 May 13;9(5):e97003. doi: 10.1371/journal.pone.0097003 (PMC4019531; doi:10.1371/journal.pone.0097003)

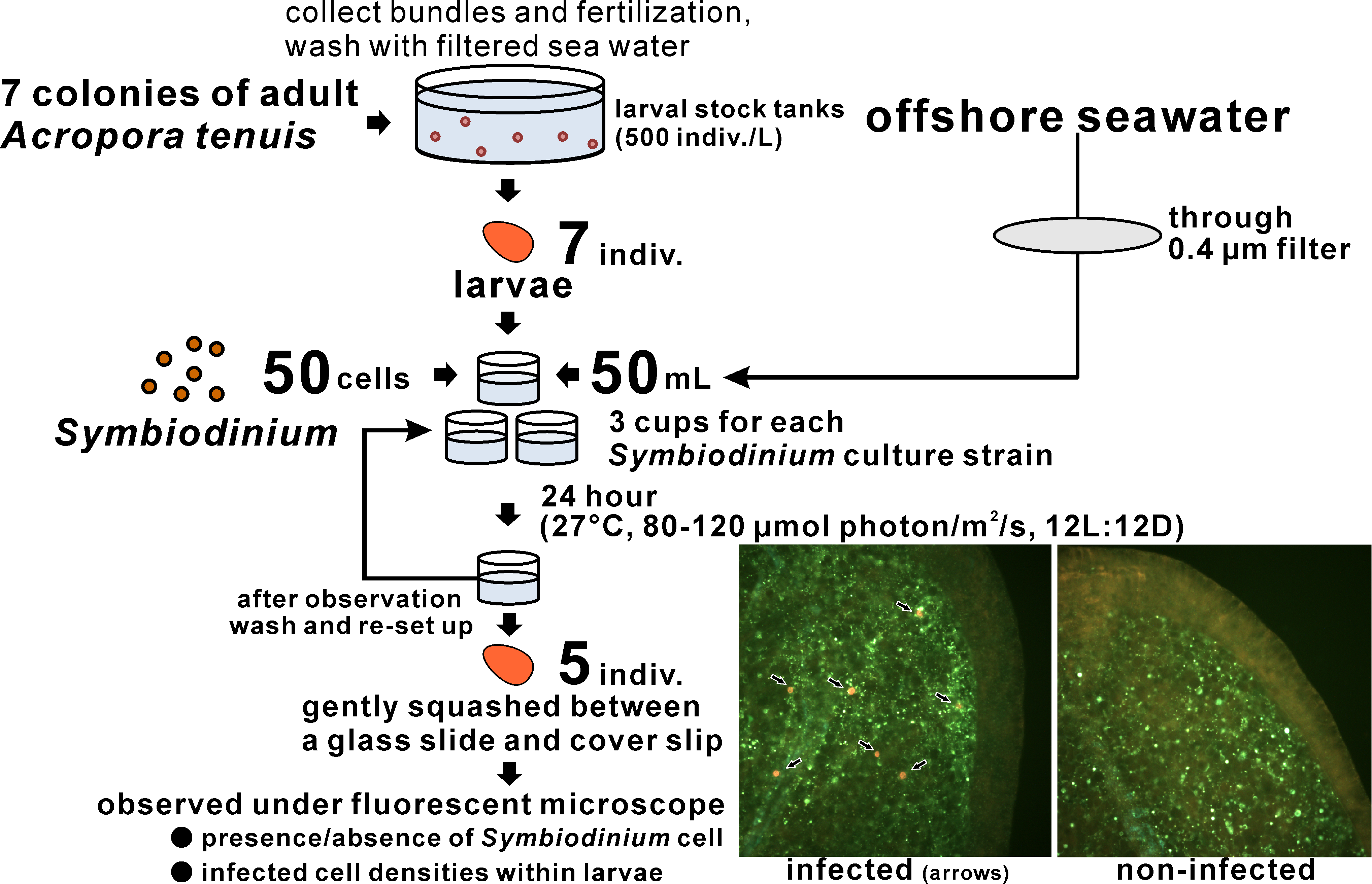

Supplement: Figure S1 — A schematic diagram of the infection test procedure. (TIF) [file pone.0097003.s001.tif]
