# Supplementary material for: Establishment of Coral–Algal Symbiosis Requires Attraction and Selection
Source: PLoS One. 2014 May 13;9(5):e97003. doi: 10.1371/journal.pone.0097003 (PMC4019531; doi:10.1371/journal.pone.0097003)
Supplement: Table S1 — Number of Symbiodinium DNA clones recovered from water and Acropora recruit samples. (DOCX) [file pone.0097003.s002.docx]

Table S1. Number of *Symbiodinium* DNA clones recovered from water and *Acropora* recruit samples.

| Water samples | Clade | | | | | | | Total | |
| --- | --- | --- | --- | --- | --- | --- | --- | --- | --- |
|  | A | | C | | D | | G |  |  |
| May 16 | 5 | | 6 | | 1 | | 12 | 24 | |
| May 17 | 0 | | 7 | | 0 | | 18 | 25 | |
| May 18 | 0 | | 0 | | 21 | | 4 | 25 | |
| May 19 | 9 | | 2 | | 11 | | 1 | 23 | |
| May 20 | 0 | | 16 | | 9 | | 0 | 25 | |
| May 25 | 5 | | 10 | | 0 | | 7 | 22 | |
| Total | 19 | | 41 | | 42 | | 42 | 144 | |
| Accession numbers | AB849693- AB849711 | | AB849712- AB849752 | | AB849753- AB849794 | | AB849795- AB849836 |  | |
|  |  | |  | |  | |  |  | |
| Recruit samples | | Species^(a)^ | | Clade | | | | | Total |
|  |  |  |  | A | | D | | |  |
| Rec11AR14_1 | | *A. nasuta/selago* | | 1 | | 7 | | | 8 |
| Rec11AR17_1 | | *A. intermedia* | | 3 | | 6 | | | 9 |
| Rec11AR18_1 | | *A. nasuta/selago* | | 0 | | 5 | | | 5 |
| Rec11AR18_2 | | *A. cytherea* | | 10 | | 0 | | | 10 |
| Rec11AR18_3 | | *A. nasuta/selago* | | 9 | | 0 | | | 9 |
| Rec11AR18_4 | | *A. divaricata* | | 8 | | 2 | | | 10 |
| Rec11AR18_5 | | *A. hyacinthus* | | 9 | | 1 | | | 10 |
| Total | | 5 species | | 40 | | 21 | | | 61 |
| Accession numbers | |  | | AB849837- AB849876 | | AB849877- AB849897 | | |  |

^(a)^The procedures used for *Acropora* recruit sample DNA extraction and species identification were reported by;

Suzuki G, Hayashibara T, Shirayama Y, Fukami H (2008), Evidence of species-specific habitat selectivity of *Acropora* corals based on identification of new recruits by two molecular markers. Mar Ecol Prog Ser 355: 149–159.
