# Supplementary material for: Establishment of Coral–Algal Symbiosis Requires Attraction and Selection
Source: PLoS One. 2014 May 13;9(5):e97003. doi: 10.1371/journal.pone.0097003 (PMC4019531; doi:10.1371/journal.pone.0097003)
Supplement: Table S2 — Symbiodinium culture strains used for infection tests are listed. (DOCX) [file pone.0097003.s003.docx]

Table S2. *Symbiodinium* culture strains used for infection tests are listed.

| Clade/Type | Strain name | Host animal |
| --- | --- | --- |
| A1^(a)^ | AJIS2-C2 | *Acropora* sp. |
| A1^(a)^ | UcM-C2 | *Acropora* sp. |
| A2 relative^(a)^ | GTP-A6-Sy | Free-living |
| A2 relative^(a)^ | ISS-C2-Sy | Free-living |
| A3^(b)^ | CS-161^(1)^ | *Tridacna derasa* |
| B^(c)^ | CCMP1633^(2)^ | *Aiptasia pulchella* |
| C^(d)^ | CCMP2466^(2)^ | *Discosoma sanctithomae* |
| D1-4^(d)^ | CCMP2556^(2)^ | *Montastraea faveolata* |
| E^(a)^ | MJa-B6-Sy | Free-living |
| F^(e)^ | CS-156^(1)^ | *Montipora verrucosa* |

Clade/type are as identified by,

^(a)^Yamashita H, Koike K (2013) Genetic identity of free-living *Symbiodinium* obtained over a broad latitudinal range in the Japanese coast. Phycol Res 61: 68–80.

^(b)^Baillie BK, Belda-Baillie CA, Maruyama T (2000) Conspecificity and Indo-Pacific distribution of *Symbiodinium* genotypes (Dinophyceae) from giant clams. J Phycol 36: 1153–1161.

^(c)^Tchernov D, Gorbunov MY, de Vargas C, Yadav SN, Milligan AJ, et al. (2004) Membrane lipids of symbiotic algae are diagnostic of sensitivity to thermal bleaching in corals. Proc Natl Acad Sci U S A 101: 13531–13535.

^(d)^LaJeunesse TC, Lambert G, Andersen RA, Coffroth MA, Galbraith DW (2005) *Symbiodinium* (Pyrrhophyta) genome sizes (DNA content) are smallest among dinoflagellates. J Phycol 41: 880–886.

^(e)^Santos SR (2004) Comment for phylogenetic analysis of a free-living strain of *Symbiodinium* isolated from Jiaozhou Bay, P.R. China. J Phycol 40: 395–397

Culture strains were purchased from;

^(1)^Commonwealth Scientific & Industrial Research Organization.

^(2)^Provasoli–Guillard National Center for Culture of Marine Algae and Microbiota.
