# Supplementary material for: Establishment of Coral–Algal Symbiosis Requires Attraction and Selection
Source: PLoS One. 2014 May 13;9(5):e97003. doi: 10.1371/journal.pone.0097003 (PMC4019531; doi:10.1371/journal.pone.0097003)
Supplement: Text S1 — Infection test using apo-symbiotic Acropora tenuis larvae and dead Symbiodinium cells. (DOCX) [file pone.0097003.s004.docx]

**Text S1.** Infection test using apo-symbiotic *Acropora* *tenuis* larvae and dead *Symbiodinium* cells.

Types A1 and D1-4 *Symbiodinium* cultures (AJIS2-C2 and CCMP2556) were readily acquired by *A. tenuis* larvae, and these *Symbiodinium* cells appeared to be attracted by the larvae (see Main Text). Therefore, as the first step in establishment of symbiosis, it may be important that *Symbiodinium* cells swim toward the larvae. To clarify this, we performed additional infection tests using dead cells of types A1 and D1-4 cultures. It is notable that live non-motile cells should be used for this experiment in normal conditions. However, we could not maintain 100% non-motile cells during the experimental period (24 h), because even non-motile cells can transform into motile cells by the next morning. Thus, we used dead cells as non-motile cells.

The experimental design was as in the Main Text, with seven individual larvae placed in glass cups with 50 mL of filtered seawater, and the cups were inoculated with 50 dead *Symbiodinium* cells that had been prepared by freezing for 24 h at −80°C. The experiment was performed in triplicates for each *Symbiodinium* culture. The same test with live *Symbiodinium* cells served as the control. Five of the seven larvae from each cup were randomly selected and observed using an epifluorescent microscope.

Infection was observed in the control cups with live *Symbiodinium* cells infection rates (average ± SD of three cups) and infected cell densities (average ± SE per infected larva) were 66.7 ± 18.9% of larvae and 2.3 ± 0.6 cells/larva and 40.0 ± 16.3% and 2.2 ± 0.6 cells/larva for types A1 and D1-4, respectively. In contrast, no infection was observed in the cups with dead *Symbiodinium* cells of either type.
